# Impact of state mandatory insurance coverage on the use of diabetes preventive care

**DOI:** 10.1186/1472-6963-10-133

**Published:** 2010-05-21

**Authors:** Rui Li, Ping Zhang, Lawrence Barker, DeKeely Hartsfield

**Affiliations:** 1 Division of Diabetes Translation, Centers for Disease Control and Prevention, Atlanta, USA; 2 The National Institute for Occupational Safety and Health, Centers for Disease Control and Prevention, Atlanta, USA

## Abstract

**Background:**

46 U.S. states and the District of Columbia have passed laws and regulations mandating that health insurance plans cover diabetes treatment and preventive care. Previous research on state mandates suggested that these policies had little impact, since many health plans already covered the benefits. Here, we analyze the contents of and model the effect of state mandates. We examined how state mandates impacted the likelihood of using three types of diabetes preventive care: annual eye exams, annual foot exams, and performing daily self-monitoring of blood glucose (SMBG).

**Methods:**

We collected information on diabetes benefits specified in state mandates and time the mandates were enacted. To assess impact, we used data that the Behavioral Risk Factor Surveillance System gathered between 1996 and 2000. 4,797 individuals with self-reported diabetes and covered by private insurance were included; 3,195 of these resided in the 16 states that passed state mandates between 1997 and 1999; 1,602 resided in the 8 states or the District of Columbia without state mandates by 2000. Multivariate logistic regression models (with state fixed effect, controlling for patient demographic characteristics and socio-economic status, state characteristics, and time trend) were used to model the association between passing state mandates and the usage of the forms of diabetes preventive care, both individually and collectively.

**Results:**

All 16 states that passed mandates between 1997 and 1999 required coverage of diabetic monitors and strips, while 15 states required coverage of diabetes self management education. Only 1 state required coverage of periodic eye and foot exams. State mandates were positively associated with a 6.3 (P = 0.04) and a 5.8 (P = 0.03) percentage point increase in the probability of privately insured diabetic patient's performing SMBG and simultaneous receiving all three preventive care, respectively; state mandates were not significantly associated with receiving annual diabetic eye (0.05 percentage points decrease, P = 0.92) or foot exams (2.3 percentage points increase, P = 0.45).

**Conclusions:**

Effects of state mandates varied by preventive care type, with state mandates being associated with a small increase in SMBG. We found no evidence that state mandates were effective in increasing receipt of annual eye or foot exams. The small or non-significant effects might be attributed to small numbers of insured people not having the benefits prior to the mandates' passage. If state mandates' purpose is to provide improved benefits to many persons, policy makers should consider determining the number of people who might benefit prior to passing the mandate.

## Background

In 2005, diabetes, both diagnosed and non-diagnosed, affected nearly 21 million Americans. The number of Americans with diabetes has risen steadily and is generally expected to further increase during the next several decades [1]. Diabetes is associated with increased risk of coronary heart disease, stroke, end-stage renal disease, amputation, and blindness. During 2007, the direct medical cost related to diabetes in the United States was 116 billion dollars [2]. Preventive care, such as self monitoring of blood glucose (SMBG), annual eye exams, and annual foot exams, can prevent or delay the complications of diabetes and may subsequently reduce the associated health care costs[3-5]. According to a 2006 national study, however, many Americans with diabetes did not perform SMBG or receive annual eye or foot exams [6].

By 2009, the District of Columbia and all states except Alabama, Idaho, North Dakota and Ohio had passed laws or regulations mandating that private insurance policies include certain benefits for diabetes, such as coverage of diabetic medication, equipment and supplies[7,8]. Although the number of state mandated benefit laws has increased dramatically since the 1990s [9], few studies have evaluated the efficacy of these mandates. For those state mandates evaluated, studies often reported limited effects, since many benefits mandated had already been covered by the insurance polices [9-11].

The purpose of our study was to evaluate the effect of the state diabetic benefit mandates on three forms of diabetes preventive care described in Healthy People 2010 objectives (SMBG, foot exams, eye exams). After gathering data on the contents of state mandates (methods described later), we hypothesized that:

(1) State mandates would have very limited national-level direct effect (through expanding number of people having covered benefits) on increasing the proportion of people with diabetes receiving annual eye exams and annual foot exams, since only one state mandated coverage of these forms of care.

(2) State mandates may have a positive direct effect on SMBG; however, the size of this effect would depend on how many insurance policies already covered diabetic monitors and strips before the mandates.

(3) State mandates could indirectly increase receipt of preventive care through increased knowledge obtained via diabetes self management education (DSME) classes, which were covered by many states' mandates.

We examined these relationships in a quantitative manner.

## Methods

### Describing the Benefits that State Mandates Covered

We gathered from state websites information on state mandates, such as benefits covered and the year the mandates were enacted. We also contacted state insurance commissioners' offices or state legislatures to confirm that the websites included all relevant data. We made a checklist of covered benefits based on the typical benefits covered in private insurance policies in the Model Contract Specifications for Services Related to Diabetes[12]. We also validated the information using National Conference of State Legislations [8] and Government Accounting Office (GAO) [13] report of state mandates on diabetes benefits. In this study, only benefits directly related to receiving SMBG, eye exams, and foot exams are considered.

### Study Population and Data Source

We obtained data on the use of diabetes preventive care, diabetes patients' demographic characteristics, and socioeconomic status from the publically available Behavioral Risk Factor Surveillance System (BRFSS). The BRFSS conducts annual, state-based, cross-sectional, random, land-based telephone surveys of 150,000-210,000 community-dwelling U.S. adults. The BRFSS survey includes a core questionnaire gathering basic information about respondents' access to care and demographic characteristics, and state-added modules for special topics, such as diabetes. All states administer the core questionnaire annually, but not all states administer the diabetes module every year [14]. We used data from 1996-2000, because relatively detailed insurance questions were asked during those years. We excluded data from states that passed mandates prior to 1996 (no pre-mandate data would be available) or passed mandates in 2000 (no post-mandate data would be available) or, if a mandate was passed, had no diabetes data either the year before the mandate, the year of the mandate, or the year after the mandate. Restricting our analyses to the 24 remaining states and District of Columbia, 16 of which passed mandates during our study period, formed a natural experiment, allowing pre- and post-mandate comparisons.

Our total sample consisted of 4,797 adults with diagnosed diabetes who had private insurance and were of age < 65 years (thus excluding the overwhelming majority of those covered by Medicare) at the time of the interview. The state median survey response rate during the study period (Council of American Survey Research Organizations' definition) varied by year and ranged from 48.9% to 63.2% [15].

### Diabetes Preventive Care Measures

We focused on three diabetes preventive care measures, derived from the national goals on diabetes in Healthy People 2010 [16]. These were: receiving a dilated eye exam within the previous year; receiving a foot exam within the previous year; and performing SMBG at least once daily. Since all these are important for diabetic patients, we also considered a fourth measure: simultaneously receiving all three.

### State Mandates Variables

We created a dichotomous variable to distinguish years before the mandate was enacted from the year the mandate was enacted and subsequent years. This variable indicates the calendar years that a mandate was/was not in place; its value varied by state. For states that did not enact a mandate, this variable was constant. This was our primary independent variable of interest, since a positive association between this variable and a given dependent variable would suggest that the mandates were effective. We did not distinguish among states by contents of mandated coverage, since almost all states' legislation mandated DSME and diabetes equipment and supplies. We also did not consider how the effects of the mandates varied among states because we wanted to estimate the average effect over all states and not simply identify within-state effects.

### Covariates

We considered both state and individual-level covariates. State covariates included: a state fixed effect to control for the state time-invariant characteristics; percentage of patients having at least one doctor visit last year (proxy for physician supply and quality of care in the state); and percentage of residents in the state with diagnosed diabetes (since states having high diabetes prevalence rate may be more likely to pass the mandates to improve the quality of care and reduce the costs of diabetes). Individual level covariates included: patient age, gender, race/ethnicity, marital status, education, income, time since diagnosis of diabetes, and insulin use.

We included 4 dichotomous variables indicating year (1997, 1998, 1999, and 2000) to account for secular trend; this controlled for the other national policies that affected the utilization of diabetes preventive care, such as The National Committee for Quality Assurance (NCQA) requirements.

### Statistical Methods

We conducted a person-level analysis. We used multivariate logistic regression models, with dependent variables: receipt of a dilated eye exam within the previous year; receipt of a foot exam within the previous year; performing daily SMBG; and all three simultaneously. We report both predicted marginal probabilities and odds ratios. We used STATA software (version 10.1, StataCorp LP 2009), accounting for the BRFSS's complex design. Results were considered significant if p < 0.05.

### Sensitivity Analyses

It is plausible that those with low income, non-Hispanic blacks, and Hispanics with insurance are more likely than others to have policies that did not cover the mandated benefits before the state mandates [17-20]; the mandates may disproportionately increase these persons' access to care. We tested for interactions between having/not having mandates and income and between mandates and membership in racial/ethnic groups. Since insulin users generally monitor blood glucose more frequently than non-users, thereby using more test strips, we did a sub-group analysis on SMBG for non-insulin users. In addition, regulations and laws may not take effect immediately. We therefore constructed a series of lag variables to capture the effect of mandates at different time after enactment: instead of one dichotomous variable, the lag variables included four dichotomous variables: the year the mandate enacted; the first year after enactment; the second year after enactment; and the third year after enactment. We tested the null hypothesis that all the four coefficients were the same.

## Results

### Description of State Mandates

Table 1 lists the states in our analysis and the year mandated insurance coverage on diabetes care benefits took effect. Sixteen states enacted mandates between 1997 and 1999 (3 in 1997; 9 in 1998, and 4 in 1999). Eight states and the District of Columbia did not have mandates as of 2000. Specific benefits covered by the mandates varied among states. Among the states with a mandate, all except Arizona mandated insurance coverage of DSME. All states mandated coverage of diabetic equipments and supplies; only Texas covered periodic eye and foot exams.

**Table 1:** Year in which mandates on diabetes took effect in the states included in the study

Year Mandated Coverage Took Effect	State
1997	Arkansas, Nevada, Tennessee,
1998	Colorado, Georgia, Kansas, Kentucky, Mississippi, New Hampshire, North Carolina^†^, Texas^†^, Vermont,
1999	Arizona, Iowa, Pensylvania, Virginia
Not having mandates as of the end of 2000	Alabama, District of Columbia^†^, Hawaii, Idaho, Michigan, Montana, North Dakota, Ohio, Wyoming

Alabama, Idaho, North Dakota, and Ohio had not passed mandates as of 2009

^†^In these states, the laws became effective later than August of the year, so in the analysis, the effective year was coded as the following year

### Characteristics of the States and the Patients

Table 2 describes the state characteristics and patient characteristics in the states that passed mandates and in the states not having mandates. The two groups of states did not differ significantly in most of the characteristics except that states that enacted mandates had: a higher proportion of Non-Hispanic Whites; a smaller proportion of the population of other race/ethnicities; and a slightly higher proportion of younger adults. The average state prevalence of diabetes was 5.5%; 30% of the people with diabetes used insulin; the mean time since detection of diabetes was 8.8 years. 90% had at least a high school education; 12% has annual family income more than $75,000 in the year prior to the interview.

**Table 2:** Comparison of state and person-level characteristics between the states that enacted mandates and those not having mandates

	States mandates enacted during 1997 and 1999 (N = 3195) Mean(95% CIs*)	States not having mandates as of 2000 (N = 1602) Mean(95% CIs*)
Rate of patients having doctoral visits last year (%)	87.8(87.6, 87.9)	87.4(87.2, 87.6)
Prevalence of diabetes in the state (%)	5.5(5.5, 5.5)	5.4(5.4, 5.5)
Insulin use among people with diabetes (%)	30.7 (29.1, 32.3)	29.2 (27.0, 31.4)
Time since diagnosis of diabetes (years)	8.7(8.4,9.0)	8.8(8.4,9.3)
Male (%)	45.9 (44.2, 47.7)	46.6 (44.2, 49.1)
Married (%)	65.7 (64.1, 67.4)	63.7 (61.3, 66.1)
Aged 18-44 y (%)	29.8 (28.2, 31.3)	26.1 (23.9, 28.3)
Aged 45-64 y (%)	70.0 (68.5, 71.6)	73.9(71.7, 76.1)
Non-Hispanic White (%)	78.9 (77.5, 80.3)	71.1 (68.9, 73.3)
Non-Hispanic Black (%)	12.4 (11.3,13.6)	12.5 (10.9,14.1)
Hispanic (%)	6.3 (5.4, 7.1)	4.7 (3.6, 5.7)
Other race/ethnicity (%)	2.4(1.9, 3.0)	11.7(10.2, 13.3)
Less than high school education (%)	10.1 (9.1,11.2)	8.7 (7.4,10.1)
High school graduate (%)	34.1 (32.5, 35.8)	32.4 (30.1, 34.7)
Some college but did not graduate (%)	28.6 (27.0, 30.2)	30.3 (28.1, 32.6)
College graduate (%)	27.1 (25.6,28.6)	28.5(26.3,30.7)
Family income <$35,000/y (%)	46.9 (45.2, 48.7)	44.3 (41.8, 46.7)
Family income $35,000-$75,000/y(%)	41.5 (39.8,43.2)	43.2 (40.8,45.6)
Family income >$75,000/y (%)	11.5 (10.4,12.7)	12.5 (10.9,14.2)

*: CIs: Confidence intervals

### Time Trends in the Utilization of Preventive Care Services at the State Level

**Figure 1:**
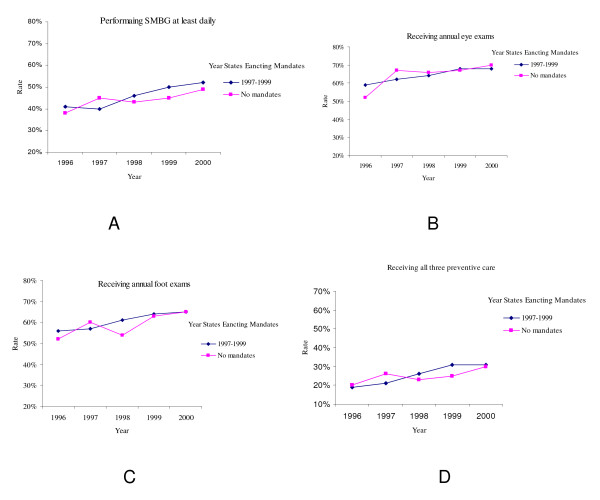
**Trends in the Rates of Receipt of Three Forms of Preventive Care in the States that Enacted Mandates in 1997, 1998, or 1999 and States not Having Mandates as of 2000**.

Figure 1 displays the unadjusted rates of diabetic patients receiving three preventive care over time in the states that enacted mandates in 1997, 1998 or 1999, and in those states not having mandates. Panels A-D show, respectively, performing daily SMBG (A), receiving annual eye exam and foot exams (B and C), and receiving all three preventive care simultaneously (D). The rates of patients performing SMBG and receiving eye and foot exams increased in both those states enacted mandates and those not having mandates except in 1998. In that year, there was a dip in the charts for all non-mandate states, while in states with mandates, the slopes were all positive. The trend lines for performing daily SMBG and receiving all three preventive care in the states that enacted mandates were slightly steeper than the corresponding lines in the states not passing mandates (trend lines not shown).

### Effects of State Mandates on the Utilization of Diabetes Preventive Care

Additional File 1 shows the predicted marginal probabilities and odds ratios of the legal variables and control variables on the use of diabetes preventive care measures considered. After controlling for the state level characteristics, patient diabetes treatment and time since diagnosis, and socio-economic and demographic characteristics, on average, state mandates were associated with: a 6.3 percentage point increase in the probability of patients performing daily SMBG (odds ratio, 1.3, P = 0.04); a 0.1 (rounded) percentage point decrease in the probability of receiving annual eye exams (odds ratio, 1.0, P = 0.92, not significant); a 2.3 percentage point increase in the probability of receiving annual foot exams (odds ratio, 1.1, P = 0.45, not significant); and a 5.8 percentage point increase in the probability of the patient receiving all three types of preventive care (odds ratio, 1.4, P = 0.03).

### State and Personal Characteristics Associated with Utilization of Diabetes Preventive Care Services

Insulin users were 40.7, 13.5, 21.3 and 27.1 percentage points (predicted probabilities) more likely to perform daily SMBG, receive annual eye exams, receive foot exams, and receive all three preventive care simultaneously, respectively. Non-Hispanic blacks were 6.2,12.5, and 6.6 percentage point more likely to receive annual eye exams, foot exams, and all three forms of preventive care. Persons who completed high school or greater had significantly higher probabilities of using all three forms of preventive care, separately or simultaneously (Additional File 1).

### Results for the Sensitivity Analysis

The coefficients of the interaction terms between mandate effect and the indicators for being non-Hispanic black or Hispanic were positive for all dependent variables, but none were significant. For the low income group, the coefficients were positive for performing SMBG, and none were significant. The coefficients of the variable indicating years after the mandates were passed in the sub-group analysis on non-insulin users were similar to the corresponding coefficients in the base case; none were significant. The null hypothesis that the variables showing the lag effects were the same was not rejected. For brevity, we do not show these specific results.

## Discussion

To the authors' knowledge, this is the first national level study of the impact of state mandatory diabetes insurance coverage on the use of diabetes preventive care. Our findings suggest that state mandates vary in their impact by preventive care type, but the impact tends to be small.

The effects of state mandates were smaller than other policies expanding insurance coverage for patients with diabetes. Li et al. analyzed the effect of the Medicare expansion on diabetes monitors and strips to non-insulin users in 1997; the probability of diabetes non-insulin users' performing daily SMBG increased by 6-16 percentage points after the expansion [21]. Karter et al. reported a 16 percentage point increase in performing daily SMBG and an 8 percentage point increase in annual eye exams among diabetes patients in a large managed care organization after it changed its reimbursement policy from no coverage to only paying a small amount of copayment for strips and eye exams [22].

One possible reason for the limited effect of mandates is that many insurance policies already covered the mandated benefits even without mandates in place; accordingly a relatively small number of people were directly benefitted. As an extreme example, a 2003 report by the Utah Insurance Department showed that, prior to the passage of Utah's 2000 law requiring insurers to cover diabetic monitors and blood testing strips, all insurance plans in the state already provided such coverage [23]. Although self-insured health plans were exempt from the mandates, a GAO report (2005) showed that the policies in 13 of the largest employers self-insured health plans covered most of the mandated benefits [13]. We are aware of no evidence that the benefit coverage of the self-insured health plans are more generous than the other private insurance policies, which the state mandates would impact. The GAO report also attributed the small number of states mandating periodical eye and foot exams to these forms of care being considered part of general health care, and therefore already covered [13].

Another possible reason for the limited effect is that unpublished work from the CDC indicates that 30%-50% of Americans with diabetes work for employers that are exempted from state mandates (self-insured organizations or the federal government); however, our data source does not allow us to identify these people. Considering the larger size of the effect of similar mandates in the Medicare population, we would probably have found larger effects here had our data sources allowed us to restrict our analyses to those whose employers were subject to state mandates and whose insurance did not cover these preventive care before the mandates [21].

The state mandates were not shown to have an effect on annual eye and foot exams, which was consistent with our hypothesis that the direct effect of state mandates would be limited. However, we also found no evidence for the hypothesized indirect effects through increased knowledge. One possible explanation is that annual eye and foot exams, unlike SMBG, require more effort from health providers than from the patients, and state mandates do not directly affect providers' practice pattern, although the mandates reduced the burden of filing paperwork claiming authorization[24]. Ceiling effects may also play a role: 63% and 59% percent of patients had received annual eye exams and foot exams before the state mandates, respectively, while only 44% performed SMBG daily. The effect of expanding insurance coverage in further increasing the proportion of patients receiving annual eye exams and foot exams might be limited. Other strategies might be considered.

Direct (mandating benefit coverage) and indirect (the effect achieved through increased knowledge obtained via DSME) effects could not be isolated, and our data did not allow us to determine whether the expanded DSME coverage actually resulted in an increase in patients taking DSME classes. All but one of the states that passed mandates between 1997 and 1999 covered DSME. DSME classes often include material concerning the importance of performing SMBG and receiving annual eye and foot exams to prevent diabetic complications [25]. Prior to the mandates being passed, coverage of DSME was unusual. For example, a study in 1994 reported that only 35.1% of the US adult population with diabetes attended any diabetes classes or programs, primarily due to lack of reimbursement [26,27]. However, our results suggest that the effects of DSME classes during the study period might be limited. This could be due to how the mandates on DSME were implemented: many mandates included restrictions on who should receive the benefits and who could be reimbursed for providing DSME [25]. Thus the benefits of mandated coverage on DSME might not be realized in the initial years after the mandates passed.

Members of racial and ethnic minorities and low income persons could plausibly be more likely than others to be underinsured, therefore, they might benefit more from the mandates. When we examined interaction terms between the variables indicating presence of a mandate and the variables indicating membership in these groups, the coefficients of the interaction terms were positive. This suggested that the state mandates might have had more effect on use of the preventive care in people who were underinsured and less likely to have these coverage before the state mandates. However, the interactions were not significant. Because these sub-groups were represented by small sample sizes, we had little power to detect interactions. Thus, it remains unknown if mandates are truly more effective in the case of underinsured persons.

Our results show that state mandates increased the probability of people with diabetes receiving all three types of preventive care simultaneously. This is important because diabetic patients need comprehensive care. From the size of the coefficients, this could plausibly be due to improved SMBG among people who received eye and foot exams. It is possible that those who did not receive annual eye or foot exams prior to the mandates (the group that most needs to be motivated to receive preventive care) were not motivated by changes in insurance coverage.

Our study has several limitations. First, our results only apply to the states included in the analysis and the three types of preventive care. The results might or might not generalize to other states and other types of care, such as DSME or prescription drugs. Second, we did not have information on how state mandates were implemented. Third, the cross-sectional nature of the data does not allow us to make causal inferences. Fourth, if there were other confounding state time-variant characteristics, our estimation might be biased. Fifth, although we used time trend to control the effects of national programs such as NCQA report cards and other diabetes quality improvement initiatives, if the effects of these programs differed among the states, our results might be confounded. Finally, our study also inherited the limitations of the BRFSS. For example, BRFSS data are self-reported; BRFSS is a land telephone-based surveillance system, excluding those without land-based telephones.

Although state mandates might have improved receipt of diabetes-related preventive care little or none, we do not advocate that mandates be repealed. Following the 1990's shrinkage of benefits covered by managed care health plans and the failure of the health care reform, state mandates were more and more used as a political force to push the problem in health care system onto the policy agenda and show that government can take action. One state's mandates can also serve as precedents for others [9]. This symbolic effect can be very powerful: during 1996-2003, 43 states and District of Columbia passed state mandates on diabetes benefits. The early state mandates may have contributed to the expansion of Medicare's coverage of diabetes in 1997 [7]. Thus the state mandates could contribute indirectly to the improvement of the diabetes care, along with the other programs such as NCQA quality report card and the state diabetes prevention and control programs. In addition, small increases in rate of utilization, over time, can add up. Since we do not know what happened after our study period, it is possible that the small increase in performing daily SMBG that we detected might, eventually, have a positive impact on public health. Furthermore, opponents of state mandates have claimed that mandates would increase insurance premiums, causing employers to drop insurance and leave more people uninsured[28]. Our analysis showed that state mandates had a small impact on using the covered benefits. Thus, it is unlikely that more than a handful of employers would drop insurance coverage due to state mandates.

## Conclusions

Our study provided the first large-scale evaluation of efficacy of state mandates on diabetes benefits. Further studies should collect data on the specific coverage provided to patients in different health plans in the states over time, and expand the health services and outcome measures considered in this study. This would support evaluation of policy effects on those that state mandates truly affected, either directly or indirectly. In addition, cost-benefit analyses, incorporating administrative costs, added costs for using the services, and the benefits from using more preventive care, should be a part of future decision making on state mandates. The primary consequence of this study (the small effects of the mandates) can be paraphrased as "small increases in benefits are likely to have small effects". If the purpose of state mandates is to provide improved benefits to many persons, state policy makers in states that do not do so should determine the number of people a mandate would benefit prior to passing the mandate. The new health care reform will expand coverage to 32 million uninsured people and mandate coverage of recommended preventive services with no cost sharing [29,30]. Following the same rationale, this could plausibly increase in the use of preventive services, particularly among the previously uninsured.

## Competing interests

The authors declare that they have no competing interests.

## Authors' contributions

RL initiated the research idea, designed the study, did the analysis, and wrote the manuscript. PZ provided valuable comments on research design and methods, and involved in writing. LB provided valuable comments on methods and helped on editing. DH compiled the state mandates information. All authors read and approved the final manuscript.

## Pre-publication history

The pre-publication history for this paper can be accessed here:

http://www.biomedcentral.com/1472-6963/10/133/prepub

## Supplementary Material

Additional file 1 **Table S3: Results of multivariate regression analysis**. The table presented estimated marginal probabilities and odds ratios of U.S. adult diabetes patients using preventive care associated with enactment of the state mandates, controlling other state and person-level characteristics. Click here for file
