# Supplementary material for: Impact of state mandatory insurance coverage on the use of diabetes preventive care
Source: BMC Health Serv Res. 2010 May 21;10:133. doi: 10.1186/1472-6963-10-133 (PMC2881060; doi:10.1186/1472-6963-10-133)
Supplement: Additional file 1 — Table S3: Results of multivariate regression analysis. The table presented estimated marginal probabilities and odds ratios of U.S. adult diabetes patients using preventive care associated with enactment of the state mandates, controlling other state and person-level characteristics. [file 1472-6963-10-133-S1.DOC]

TABLE S3. Results of multivariate analyses:

estimated marginal probabilities and odds ratios of U.S. adult diabetes patients using preventive care associated with enactment of the state mandates and other state and person-level characteristics^†&^

|  | **Daily SMBG** | **Annual eye exams** | **Annual foot exams** | **Receiving all three** |
| --- | --- | --- | --- | --- |
| Legal variables |  |  |  |  |
| After state mandate took effect | 0.063*  1.3 (1.0-1.7) | -0.0005  1.0(0.8-1.3) | 0.023  1.1(0.9-1.4) | 0.058*  1.4(1.0-1.9) |
| Time trends |  |  |  |  |
| 1997 | 0.014  1.1(0.8,1.4) | 0.026  1.1(0.9,1.4) | 0.011  1.0(0.8,1.3) | 0.042  1.3(0.9,1.7) |
| 1998 | 0.002  1.0(0.7,1.4) | 0.011  1.1 (0.8,1.4) | -0.021  0.9(0.7,1.2) | 0.016  1.1(0.8,1.6) |
| 1999 | 0.028  1.1(0.8,1.7) | -0.003  1.0(0.7,1.5) | -0.008  1.0(0.7,1.4) | 0.012  1.1(0.7,1.7) |
| 2000 | 0.062  1.3(0.8,2.1) | 0.004  1.0(0.7,1.6) | -0.002  1.0(0.6,1.5) | 0.012  1.1(0.6,1.8) |
| State characteristics |  |  |  |  |
| Rate of patients having doctor visits last year (%) | 0.004  1.0(1.0,1.1) | -0.0002  1.0(1.0,1.0) | 0.002  1.0(1.0,1.0) | 0.0005  1.0(1.0,1.0) |
| State diabetes prevalence rate (%) | 0.038  1.1(0.9,1.5) | 0.053  1.2(1.0,1.6) | 0.045  1.2(0.9,1.6) | 0.056*  1.4(1.0,1.9) |
| Patient characteristics |  |  |  |  |
| Using insulin | 0.407***  5.8(4.9,6.7) | 0.135***  1.9(1.6,2.2) | 0.213***  2.6(2.2,3.1) | 0.271***  4.0(3.4,4.7) |
| Years since diagnosis of diabetes | -0.002  1.0(1.0,1.0) | 0.003***  1.0(1.0,1.0) | -0.001  1.0(1.0,1.0) | 0.002**.  1.0(1.0,1.0) |
| Male | -0.058**  0.8(0.7,0.9) | -0.021  0.9(0.8,1.0) | 0.004  1.0(0.9,1.1) | -0.025*  0.9(0.8,1.0) |
| Married | -0.003  1.0(0.9,1.1) | 0.009  1.0(0.9,1.2) | 0.011  1.0(0.9,1.2) | 0.004  1.0(0.9,1.2) |
| Age between 45-64 | 0.001  1.0(0.9,1.2) | 0.111***  1.6(1.4,1.8) | 0.078***  1.4(1.2,1.6) | 0.037**  1.3(1.1,1.5) |
| Non-Hispanic Black | -0.019  0.9(0.8,1.2) | 0.062**  1.3(1.1,1.6) | 0.125***  1.8(1.4,2.2) | 0.066**  1.5(1.1,1.9) |
| Hispanic | -0.060  0.8(0.6,1.0) | 0.028  1.1 (0.9,1.5) | 0.034  1.2(0.9,1.5) | -0.032  0.8(0.6,1.1) |
| Other racial/ethnical groups | -0.057  0.8(0.6,1.1) | -0.009  1.0(0.7,1.4) | -0.032  0.9(0.6,1.2) | -0.083*  0.6(0.4,0.8) |
| High school | 0.066*  1.3(1.0,1.6) | 0.055*  1.3(1.0,1.6) | 0.025*  1.1(0.9,1.4) | 0.065*  1.4(1.1,1.9) |
| Some college | 0.121***  1.6(1.3,2.1) | 0.097***  1.6(1.2,1.9) | 0.055*  1.3 (1.0,1.6) | 0.097**  1.7(1.3,2.3) |
| College graduates or above | 0.082**  1.4(1.1,1.8) | 0.119***  1.7(1.4,2.2) | 0.058  1.3(1.0,1.6) | 0.123***  2.0(1.4,2.6) |
| Income between$35k and 75k | 0.006  1.0(0.9,0.2) | 0.008  1.0(0.9,1.2) | 0.005  1.0(0.9,1.2) | 0.002  1.0(0.9,1.2) |
| Income above $75k | 0.012  1.1(0.8,1.3) | 0.056*  1.3(1.0,1.6) | 0.014  1.1(0.9,1.3) | 0.020  1.1(0.9,1.4) |

^†^ using state fixed effect

^&^ Numbers in the upper level of each cell is average marginal effect; numbers in the lower level of each cell is odds ratio and 95% Confidence Intervals

* *p* ≤ 0.05; ^**^ *p* ≤ 0.01; ^***^ *p* ≤ 0.001
